# Keeping sight of copper in single-atom catalysts for electrochemical carbon dioxide reduction

**DOI:** 10.1038/s41467-022-30027-x

**Published:** 2022-04-27

**Authors:** Charles E. Creissen, Marc Fontecave

**Affiliations:** Laboratoire de Chimie des Processus Biologiques, CNRS UMR 8229, Collège de France, Paris, France

**Keywords:** Electrocatalysis, Catalytic mechanisms, Electrocatalysis, Electrocatalysis, Electrocatalysis

## Abstract

Cu-based single atom catalysts can convert CO_2_ into multi-carbon products, however, the assignment of active sites needs great caution. In this comment, the authors discuss the transient Cu cluster formation as active sites and emphasise the need for operando characterisation in mechanistic study.

## Teaser

Carbon dioxide can be electrochemically converted into valuable multi-carbon products using Cu-based single-atom catalysts. However, transient cluster formation, which is undetectable using ex-situ techniques, may be responsible for C_2+_ products. Here we discuss these observations to highlight the need for *operando* characterisation when defining active sites.

The advent of single-atom catalysts (SACs) extended the range of available materials for a variety of catalytic reactions^1^. Support-anchored isolated metal sites benefit from high atom efficiency with reduced material costs, and provide scope for rational modification analogous to molecular complexes^2^. In electrochemical CO_2_ reduction (CO_2_R), a technology capable of using renewable energy to generate carbon-based fuels^3,4^, Cu-based single-atom catalysts (Cu-SACs) are particularly interesting due to their ability to generate C_2+_ products with high selectivity. Consequently, structural features have been used to explain activity and selectivity trends using combined experimental and computational methods. However, observations of alterations to active sites that can occur under operating conditions have complicated mechanistic understanding. Here, we use specific examples to emphasise the need for caution when assigning the active site structure to aid the development of Cu-SACs for CO_2_R.

## Carbon dioxide reduction at Cu single-sites

**Fig. 1:**
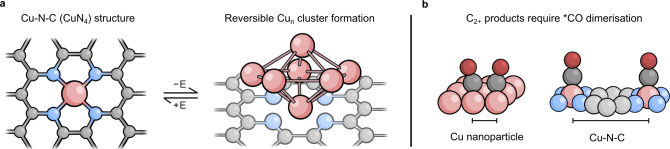
Reversible agglomeration and CO dimerisation mechanism. **a** Schematic showing the formation of Cu_n_ clusters under applied cathodic potential (−E) and the reversibility under oxidative potentials. **b** Illustration of how Cu–Cu distances in Cu-N-C materials are not compatible with *CO dimerisation.

SACs can incorporate a range of metals, heteroatoms, and supports. The most widely used examples for CO_2_R employ N-doped conductive carbon supports with N-chelated metal sites, termed M-N-C catalysts (Fig. 1a). Although reaching sufficient metal loading required for high reaction rates without the formation of metal aggregates remains a significant challenge^5^, M-N-C catalysts have shown good selectivity for CO_2_R at low overpotentials (<0.6 V)^6,7^. These catalysts are particularly interesting as they share common ground with molecular catalysts such as metal-coordinated phthalocyanines, porphyrins, and polypyridyl complexes, which are acclaimed for their high selectivity in CO_2_R^8^. In M-N-C materials, active sites are often less distinct, with mixtures of pyrrolic, pyridinic, porphyrin-like, and graphitic M-N moieties existing in parallel, but specific ratios can be tuned by synthetic procedures and modifications, offering routes to structural control.

Cu-SACs have gained interest due to their ability to convert CO_2_ into C_2+_ products with high selectivity. Therefore, detailed investigations have employed a wide range of characterisation techniques to identify specific performance-enhancing structural features. For Cu-N-C materials, the catalyst structure can be determined ex situ before and after electrolysis to observe any irreversible modifications. For example, X-ray photoelectron spectroscopy (XPS) can give the ratio and predominant nature of the nitrogen environments, high-angle annular dark-field scanning transmission electron microscopy (HAADF-STEM) can detect the presence of isolated sites, and X-ray absorption spectroscopy (XAS) can identify oxidation states with X-ray absorption near edge structure (XANES) and coordination environment with extended X-ray absorption fine structure (EXAFS) analysis. These techniques among others can provide a structural basis for computational modelling to determine possible reaction mechanisms and derive structure-function relationships. However, dynamic alterations that are only observable under working conditions have raised concerns over the true nature of the active site.

## Transient structural alterations

Despite ex situ analysis showing that Cu-N-C single sites are retained after electrolysis, *operando* spectroscopic techniques have identified that the cathodic applied potentials required for CO_2_R can cause reduction of Cu^2+^ sites to Cu^0^, which decreases the affinity for N-coordination and gives rise to Cu agglomeration (Fig. 1a). Such an effect was observed in a Cu-N-C material composed of predominantly Cu-N_4_ sites, which exhibited high ethanol selectivity (faradaic efficiency for ethanol (FE_EtOH_) = 55% with gas recirculation)^9^. Ex situ characterisation using XPS, HAADF-STEM, and XAS showed no signs of transformation, with the same structural motifs observed pre- and post-electrolysis. However, *operando* XAS at potentials below −0.6 V vs. RHE showed Cu^2+^ reduction to Cu^0^ and Cu–Cu coordination signals in XANES and EXAFS spectra respectively, signifying the formation of subnanometer Cu clusters. Upon exposure to air, or through the application of oxidative potentials, the Cu–N_4_ signals of the starting material were fully recovered while the Cu–Cu signal disappeared, showing that nanocluster formation within the Cu-N-C material is reversible. In other words, re-oxidation of the Cu clusters causes re-dispersion of the Cu atoms into N-chelated single-sites. The reversibility of the process precludes characterisation with ex situ techniques, therefore *operando* techniques are required to observe these dynamic changes.

Several subsequent systems employing *operando* techniques have displayed similar behaviour, which is not restricted to solely Cu-N-C materials. A recent study on oxygen-ligated isolated Cu^2+^ sites also gave high ethanol yields (FE_EtOH_ = 91%)^10^. Again, where ex situ characterisation showed no structural changes, *operando* XAS data were able to link the high selectivity to the reversible emergence of Cu_*n*_ clusters. The same observations of cluster formation have also extended to isolated Cu sites confined in a covalent triazine framework for C_2+_ product generation^11^. The absence of Cu ions in solution after application of an anodic potential further confirms that the Cu clusters can dissociate to re-anchor Cu atoms as single-sites within the framework. These examples suggest that C_2+_ products could be an indicator of agglomeration. The mechanistic requirements for forming multi-carbon species provide some insights into why this may be the case.

Extensive studies on bulk and nanoparticulate catalysts have shown that C–C coupling involves the dimerisation of surface-bound *CO intermediates^12^. The large interatomic distances typically observed for Cu-N-C catalysts would not allow for this process to take place at single-sites, implying that either an entirely different mechanism is taking place or that reversible restructuration occurs under working conditions (Fig. 1b). Therefore when C_2+_ products are observed, cluster formation is highly probable. Recent examples where multi-carbon products were generated from Cu-SACs have suggested different mechanistic explanations. Studies have proposed that CO–CO coupling can occur at single sites^13,14^, or that Cu sites can be sufficiently close in distance to facilitate dimerisation^15^. However, more in-depth characterisation is required to definitively rule out agglomeration and validate these assumptions.

Although agglomeration likely accounts for multi-carbon products, C_1_ products can also be formed when clusters are present. In one study, a Cu-polyphthalocyanine catalyst underwent aggregation to form nanoparticles under operating conditions, but the catalyst only generated CO^16^. Adjacent Cu sites on Pd_10_Te_3_ nanowires also generated CO and not C_2+_ products^17^. Pre-formed clusters have also produced CH_4_ or HCOOH as the sole carbon product from CO_2_R, showing that clusters do not always form C_2+_ products^18,19^. In essence, although C_2+_ products are a likely symptom of agglomeration, they are not a sole indicator and *operando* techniques are necessary to confirm the structure of the active site. Consequently, researchers should be careful when assessing the true nature of the active site to avoid the pursuit of incorrect rational modification routes.

## Understanding reversible cluster formation

SACs incorporating metals other than Cu can also undergo agglomeration, however, the degree of M^0^ formation is typically much lower than that of Cu-SACs and often not reversible^20,21^. Therefore, the possible causes behind cluster formation and the reversibility of this process must be understood through assessment of specific Cu-based examples. The dynamic behaviour arises from the combined effects of the metal coordination environment and metal-support interactions.

CO_2_R requires electrochemical reduction of Cu sites in Cu-N-C catalysts, which decreases their affinity for the support, facilitating atom migration and cluster formation. Consequently, avoiding agglomeration may simultaneously prevent catalysis. Interestingly, the clusters formed by restructuration are highly selective for C_2+_ products and are therefore beneficial to selectivity. Low-coordination Cu-based species take advantage of this unexpected form of structure-function relationship, however synthetic methods for cluster formation are typically complex^18,19^. In situ electrochemical synthesis offers a straightforward alternative route to cluster formation, in which stabilisation methods through support interactions can be used to control the cluster size to target specific products^22,23^. The role of the support in reversible cluster formation is well-illustrated by comparison with molecular catalysts. Solution-based molecular complexes are susceptible to potential-dependent decomposition, where reduction of the metal centre can increase ligand lability and promote irreversible heterogeneous material deposition, as demonstrated by deposition of Co from cobaloximes and Cu from Cu-cyclam^24,25^. However, with copper (II) phthalocyanine (CuPc) supported on carbon-nanotubes (CNTs) through π–π stacking, cluster formation was reversible as with the Cu-N-C examples^26^. In the same study, a supported porphyrin also showed reversible agglomeration whereas a Cu-cyclam, which does not anchor well to the CNTs, showed irreversible decomposition. Additionally, the reversibility of the process is not only driven by the high affinity of N sites for Cu(II), but is also dependent on particle size where smaller clusters are more easily reverted to atomic sites^27^.

The added understanding of structural attributes from *operando* analysis has opened new routes to selective CO_2_R. Up to now, *operando* XAS has proven the most applicable technique to study restructuration, but the absence of M–M signals does not always signify the absence of clusters due to problems associated with disorder and interference^28^. Therefore, alternative verification methods would be desirable. However, the range of available spectroscopic techniques that can observe such changes *in-operando* is still limited. In situ infrared and Raman spectroscopy are capable of indirectly distinguishing between clusters and single-sites due to differences in signals arising from surface-bound molecules^29^. However, analysis is complicated by dynamic desorption and adsorption of species under electrochemical conditions. The development of emerging in situ spectroscopic techniques such as ambient pressure XPS (AP-XPS) and liquid cell TEM (LC-TEM) could extract more information about the reversible process and identify active structures to provide computational methods with a correct basis to explore reaction mechanisms^30^. Innovative solutions coupling multiple *operando* techniques will likely prove vital in revealing the true nature of active sites.

## Outlook

We have highlighted that reversible cluster formation occurs in Cu-SACs under CO_2_R conditions. The in situ formation of Cu clusters can give rise to higher C_2+_ selectivity than conventional Cu catalysts, opening straightforward synthetic routes to valorise CO_2_R. However, the future of SACs for CO_2_R relies on validation of cluster formation to define structure-activity relationships that can guide rational catalyst development. We, therefore, reiterate that *operando* techniques should be commonplace when using Cu-SACs for CO_2_R due to the prevalence of reversible restructuration.
